# You Have Received More Help than I Did and I Envy You: A Social Comparison Perspective on Receiving Help in the Team

**DOI:** 10.3390/ijerph19148351

**Published:** 2022-07-08

**Authors:** Shaoqin Han, Yuanfang Zhan, Lu Zhang, Renyan Mu

**Affiliations:** 1 School of Entrepreneurship, Wuhan University of Technology, Wuhan 430070, China; hanshaoqin@hotmail.com; 2 School of Economics and Business Administration, Central China Normal University, Wuhan 430070, China; 3 School of Management, Wuhan University of Technology, Wuhan 430070, China; zhanglu9393@whut.edu.cn (L.Z.); murenyan@whut.edu.cn (R.M.)

**Keywords:** upward social comparison of received help, envy, interpersonal citizenship behavior, social comparison orientation

## Abstract

In the current research, we developed and tested a model of how and when upward social comparison of received help influenced an employee’s interpersonal citizenship behavior. Based on social comparison theory, we posited that upward social comparison of received help triggered an employee’s feelings of envy, which in turn had a negative relationship with interpersonal citizenship behavior (ICB). Further, we argued that the effects of upward social comparison of received help on envy differed in the employee’s social comparison orientation. Using data collected in three waves from 411 employees in China, we found that upward social comparison of received help was positively associated with the employee’s feelings of envy while controlling for overall receiving help, which further negatively affected interpersonal citizenship behavior. Moreover, the relationship between the upward social comparison of received help and the employee’s feelings of envy was stronger when employees had high levels of social comparison orientation and further strengthened the indirect relationship between the upward social comparison of received help and the employee’s ICB via envy. Overall, these findings have the potential to extend our knowledge of the adverse effects of receiving help in a team by introducing a social comparison perspective.

## 1. Introduction

Interpersonal helping behavior within an organization has been described as the glue that binds an organization together [1]. Helping behavior in the organization is conducive to improving organizational performance and building harmonious interpersonal relationships [2]. In general, the positive results of helping behavior are indirectly produced by employees receiving help [3], and these positive effects are typically explained using social exchange theory [4]. Specifically, receiving help reflects the goodwill and support of helpers, thereby likely triggering positive feelings of gratitude [5] and engagement in more interpersonal citizenship behaviors [6]. 

However, this stream of research ignores the specific context of the organization. Nowadays, more and more organizations work in teams, which makes receiving help observable and comparable [7]. Social comparison theory [8] indicates that employees are inclined to make social comparisons at the workplace and direct their attitudes and behaviors to their jobs [9,10]. Since receiving help can gain relevant resources such as instrumental help and emotional help, which is conducive to relieving work pressure and improving performance [2], employees are likely to make an upward social comparison, that is, compared with employees who get more help in the team. 

Given the social context in which receiving help occurs [3], a pertinent question is whether the positive effect of receiving help will still exist when employees conduct upward social comparison. Many studies have found that upward social comparison can activate the emotion of envy [11,12], which is a negative emotion that individuals experience when they are in a disadvantageous position in resource competition. Therefore, this upward social comparison of received help may evoke envy, thus reducing their citizenship behaviors to preserve their advantages and resources. 

**Figure 1:**
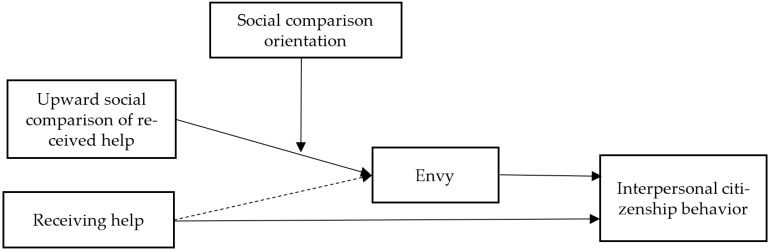
The theoretical model.

Additionally, researchers have suggested that there may be crucial individual differences in how they make social comparisons [13,14]. Hence, in the current research, we draw upon social comparison theory to explore the boundary role of employees’ social comparison orientation, which refers to individuals’ sensitivity to the behavior of others and the degree of uncertainty about the self [14]. Compared with those who receive more help in the team, employees with higher social comparison orientation stimulate stronger feelings of envy due to the perception of unfair distribution of resources [15]. As such, we posit that social comparison orientation would strengthen the positive effects of upward social comparison of received help on the employee’s emotions and behaviors. Figure 1 depicts the theoretical model.

In examining these issues, the current study makes three contributions to the literature. First, this study adopts the new perspective of social comparison [8] to explore the impact of upward social comparison of received help on employees’ psychology and behavior in the team. It further expands and supplements the previous research on the effects of employees’ absolute value of received help based on social exchange theory [4]. Second, we extend the knowledge of the negative consequences of receiving help in the specific context of the team. By exploring envy as an underlying psychological mechanism that helps explain the influence of upward social comparison of received help on employees’ workplace outcomes, we challenge the assumption that all employees will repay received preferential treatment [16]. Third, by examining the moderating effects of social comparison orientation on the relationship between the upward social comparison of received help and feelings of envy, we also extend the boundary condition of the employee’s personality characteristics in the process of receiving help and social comparison [17]. 

## 2. Theory and Hypotheses

### 2.1. Upward Social Comparison of Received Help and Employee’s Envy

Previous studies have discussed the influence of the absolute extent of receiving help drawn from social exchange theory [4]. However, social comparison theory [8] indicates that employees are inclined to make social comparisons at the workplace and direct their attitudes and behaviors to their jobs [9,10]. Among them, the most likely is to make an upward comparison, that is, with people who perform better than themselves or those who obtain more resources [11,12]. As more and more modern organizations collaborate in teams, interaction and helping behaviors among colleagues are more common and comparable [7]. Receiving more help to a great extent means gaining more resources and obtaining better performance, and employees are likely to make an upward comparison of received help. 

When employees make an upward comparison of received help, they are likely to engender envy due to the perception of unfair resource allocation [15]. Specifically, for those team members who receive more help, it means that they obtain richer resources such as care, encouragement, and sympathy, which can help them recover from negative emotional states [18]. These resources are tangible and specific, which can directly help the recipients promote various jobs and tasks or improve their work status and reduce their work pressure [2]. Moreover, the resources in the team are limited, and others’ access to more resources and performance may pose a status threat to themselves [19]. Thus, we propose the following hypothesis:

**Hypothesis** **1.** *Upward social comparison of received help is positively related to the employee’s feeling of envy, controlling for overall received help.*

### 2.2. Upward Social Comparison of Received Help, Envy, and Interpersonal Citizenship Behavior 

According to social comparison theory, people compare themselves with others to define and evaluate the self, reduce uncertainty, and seek self-enhancement [13]. Upward comparisons give rise to feelings of inferiority often associated with envy [20]. Envy surfaces when a person “lacks another’s superior quality, achievement, or possession and either desires it or wishes that the other lacked it” [21]. Empirical research has revealed that envy includes negative feelings of resentment and a desire to pull the other person down [22] because envy is a contrastive emotion that leads employees to focus on the gap between themselves and others [20]. 

Social comparison theory suggests that emotions play a central role in how individuals process social comparisons [23]. Hence, envy is a strong predictor of employee subsequent behavior [24]. In particular, employees who make an upward social comparison of received help are motivated to alleviate the emotion of envy and reduce the gap between themselves and the envied person [25]. Therefore, the most direct way for employees to narrow the gap with those who receive more help is to reduce their interpersonal citizenship behavior. Interpersonal citizenship behavior (ICB) is a specific type of organizational citizenship behavior (OCB) and is seen as a large range of prosocial behaviors, which include person-focused help and task-focused help (e.g., interpersonal helping, altruism, interpersonal facilitation) [26]. 

Specifically, envy motivates them to narrow the gap to maintain balance. This experience is so frustrating that the envious are motivated to restore the balance. However, because there are sanctions against open expressions of envy in the workplace [21], people tend to use covert means to bring down the envied targets to restore their psychological balance [25]. Reducing interpersonal citizenship behavior will not only reduce the resources obtained by others but also reduce the loss of their own resources and time because helping behavior itself is a thing that consumes resources [27]. Therefore, employees who have received less help relatively may intentionally reduce interpersonal citizenship behavior toward their peers out of envy. 

As previously mentioned, upward social comparison of received help is likely to be a significant predictor of envy, such that when employees are aware that they have received help less than others, they will elicit stronger feelings of envy. Such feelings will, in turn, negatively shape their ICB. Thus, we propose the following hypothesis:

**Hypothesis** **2a.** *The feeling of envy is negatively related to the employee’s interpersonal citizenship behavior.*

**Hypothesis** **2b.** *The feeling of envy will mediate the relationship between the upward social comparison of received help and the employee’s interpersonal citizenship behavior.*

### 2.3. The Moderating Role of Social Comparison Orientation

According to social comparison theory, there may be crucial individual differences in the frequency and extent to which individuals make social comparisons [13,14]. The social comparison orientation (SCO) describes such individual differences. In the current research, we argue that social comparison orientation, a stable individual tendency, might moderate the relationship between the upward social comparison of received help and employees’ feeling of envy. 

An individual high in social comparison orientation is “sensitive to the behavior of others and has a degree of uncertainty about the self, along with interest in reducing self-uncertainty” [14]. When employees with a high social comparison orientation perceive that their colleagues have obtained more tangible and intangible help and resources, they will be more sensitive to the status threat and respond with stronger emotions when taking information about others. Therefore, compared with those employees with lower social comparison orientation, upward social comparison of received help will be more likely to elicit the employee’s feeling of envy. Thus, we propose the following hypothesis:

**Hypothesis** **3.** *Social comparison orientation will moderate the relationship between the upward social comparison of received help and the employee’s feeling of envy, such that this relationship will be positive when social comparison orientation is high (vs. low), controlling for overall received help.*

Combining the moderating role of social comparison orientation and the mediating role of envy results in moderated mediation models, and we propose the following hypothesis:

**Hypothesis** **4.** *The indirect relationship between the upward social comparison of received help and the employee’s ICB,* via *the feeling of envy, is more negative when social comparison orientation is high (vs. low), controlling for overall received help.*

## 3. Methods

### 3.1. Participants and Procedure

To test our theoretical views, we built our surveys on Wenjuanxin (at www.wjx.cn, accessed on 1 March 2021), a reliable Chinese data collection platform similar to Qualtrics and used in many previous studies [28]. To qualify, participants should work in a team environment such that relatively frequent interactions in the team context help to accelerate social comparing processes. To reduce the potential impact of common method variance [29], we used a multi-wave design and separated our measures at different time points. At Time 1, 637 participants completed a survey that included items on employees’ upward received help social comparison, receiving help, social comparison orientation, and demographic information (age, gender, organization tenure, educational background). One month later, 509 respondents who completed the first-time survey completed the second survey of envy. Employees completed scales of ICB four weeks later at Time 3, and 416 participants completed this survey. 

Three-stage data matching was carried out through the employee’s number, and invalid questionnaires with incomplete answers and failed attention tests were eliminated. The final sample consisted of 411 employees from multiple industries, including hospitality, banking, manufacturing, communications, and education. Their average age was 31.69 years (*SD* = 4.96), and 215 (52.4%) were female. In terms of education level, six participants held a doctoral degree (1.5%), 38 had a master’s degree (9.3%), 200 had a bachelor’s degree (48.8%), and 166 had a high school education or below (40.5%). Participants’ average tenure within their current organization was 5.36 years (*SD* = 2.92). Participants were compensated with RMB 80 (about USD 12) for completing both three-stage questionnaires.

### 3.2. Measures

We translated the original English items into Chinese following a back-and-forth translation procedure to ensure their accuracy. More details of our measurements are presented below.

**Upward social comparison of received help.** We measured upward social comparison of received help using three items adapted from the receiving help scale developed by Uy et al. (2017) [2]. Employees were asked to recall the colleagues who received more help than them in the team and indicate the extent to which these colleagues received more help at work (1 = “strongly disagree”; 7 = “strongly agree”). An example is “Compared with me, these colleagues received more help in their work tasks in our department (team)” (α = 0.89).

**Envy.** We measured envy using the five-item scale developed by Duffy et al. (2012) [25]. An example is “It is somewhat annoying to see others have all the luck in getting the best assignments” (1 = “strongly disagree”; 7 = “strongly agree”; α = 0.84).

**ICB.** We measured ICB using the eight-item scale developed by Settoon and Mossholder (2002) [26]. An example is “I take time to listen to coworkers’ problems and worries” (1 = “strongly disagree”; 7 = “strongly agree”; α = 0.95)**.**

**Social comparison orientation.** Social comparison orientation was measured using the four-item scale developed by Gibbons and Buunk (1999) [14]. An example is “I always pay a lot of attention to how I do things compared with how others do things” (1 = “strongly disagree”; 7 = “strongly agree”; α = 0.93).

**Controls.** We controlled for employees’ demographics in the analysis model, including gender (1 = male, 2 = female), age (years), organization tenure (years), and educational background (1 = high school or below degree, 2 = bachelor’s degree, 3 = master’s degree, 4 = doctoral degree). Furthermore, to determine whether upward received help social comparison affects envy above receiving help, we controlled for receiving help using the three-item receiving help scale [2]. A sample item was “My coworker went out of his/her way to help me” (1 = “never”; 7 = “always”; α = 0.82). The results of the model without controls were not significantly different from the model with controls, and we include these variables in our following analysis.

### 3.3. Analytic Strategy

Employing Mplus 8.3 (Muthén & Muthén, 1998–2019), we conducted path analysis, where we simultaneously modeled all focal variables from Figure 1 along with our controls to test Hypothesis 1 (the effect of upward social comparison of received help on envy), Hypothesis 2a (the effect of envy on ICB), and Hypothesis 3 (the interaction effect of upward social comparison of received help and social comparison orientation on envy). To test Hypothesis 2b (the indirect effects of upward social comparison of received help on ICB through envy) and Hypothesis 4 (the moderated mediation model), we utilized bootstrapping to estimate the significance of indirect effects [30], as it estimates Type I error rates more accurately and is more powerful than traditional mediation tests. Further, to examine the moderation effect of social comparison orientation, our work applied grand-mean centering for social comparison orientation and upward social comparison of received help. We also grand-mean centered all other predictors.

## 4. Data Analysis and Results

### 4.1. Descriptive Statics and Correlations

Table 1 presents the means, standard deviations, and correlations among our variables.

### 4.2. Factor Analysis

Before examining the hypotheses, we conducted a confirmatory factor analysis to test the distinctiveness of our focal variables, which included receiving help, upward social comparison of received help, envy, ICB, and social comparison orientation. Results revealed that the five-factor model had satisfactory fit (*χ*^2^ = 306.11, *df* = 109, *p* < 0.001; standardized root mean square residual (SRMR) = 0.05; root mean square error of approximation (RMSEA) = 0.07, comparative fit index (CFI) = 0.95, Tucker–Lewis index (TLI) = 0.94) and fit the data significantly better than alternative models. In general, these results were encouraging with respect to the discriminant validity of our focal variables. 

### 4.3. Hypothesis Testing

The results of path analysis are displayed in Table 2. Upward social comparison of received help significantly predicted the feeling of envy (*γ* = 0.29, *SE* = 0.05, *p* < 0.001), so Hypothesis 1 is supported. The feeling of envy negatively relates to employees’ interpersonal citizenship behavior (*γ* = −0.13, *SE* = 0.05, *p* < 0.01), so Hypothesis 2a is supported. We used bootstrapping analysis with 20,000 iterations to test indirect effects. Results revealed that the indirect effect of upward social comparison of received help on ICB via envy is significant (*γ* = −0.04, 95% CI = [−0.073, −0.011]), as the 95% confidence interval for the indirect effect did not include zero, so Hypothesis 2b is supported. 

**Figure 2:**
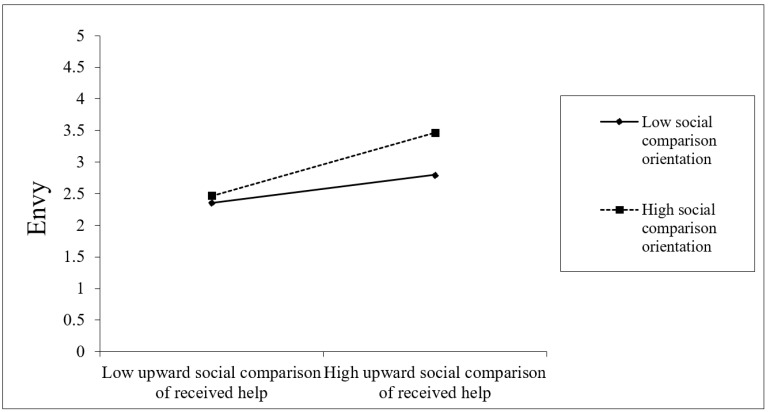
Moderating effect of social comparison orientation on the relationship between upward social comparison of received help and envy.

Hypothesis 3 predicted that social comparison orientation would strengthen the effect of upward social comparison of received help on envy, such that the effect of upward social comparison of received help on envy is stronger for individuals higher (vs. lower) in social comparison orientation. The interaction between upward social comparison of received help and social comparison orientation shown in Table 2 was significant (*γ* = 0.12, *SE* = 0.05, *p* < 0.05). To facilitate the interpretation of this interaction effect, we performed simple slope analyses [30] and examined the effect of upward social comparison of received help on envy at two conditional values of social comparison orientation (+1 *SD* and −1 *SD*). The effect of upward social comparison of received help on envy was more significantly positive (*γ* = 0.40, *SE* = 0.07, *p* < 0.001) when social comparison orientation was high (at +1 *SD*), and less positive (*γ* = 0.19, *SE* = 0.06, *p* < 0.01) when social comparison orientation was low (at −1 *SD*) (diff = 0.21, 95% CI = [0.03, 0.38]). Hypothesis 3 is thus supported. The interaction pattern is depicted in Figure 2.

**Figure 3:**
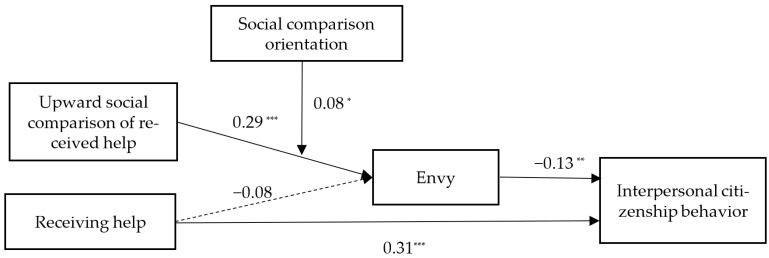
Unstandardized estimates of path coefficients. * *p* < 0.05; ** *p* < 0.01; *** *p* < 0.001.

Hypothesis 4 predicted that social comparison orientation would moderate the indirect effects of upward social comparison of received help on ICB via envy. We used bootstrapping analysis with 20,000 iterations to test these conditional indirect effects. Specifically, in support of Hypothesis 4, the indirect effect between the upward social comparison of received help and ICB through envy was more negative when social comparison orientation was high (*γ* = −0.05, 95% CI = [−0.10, −0.02]) and less negative when social comparison orientation was low (*γ* = −0.03, 95% CI = [−0.06, −0.01]); moreover, the difference between these indirect effects was significant (diff = −0.02, 95% CI = [−0.07, −0.01]). Figure 3 presents parameter estimations for this path analytical model.

## 5. Discussion

Using a three-wave survey study, we explored the social comparison perspective to understand the effects of receiving help. In line with social comparison theory, our results suggested that upward social comparison of received help triggers employees’ feelings of envy and reduces subsequent interpersonal citizenship behavior, even controlling their absolute value of received help. Moreover, our findings suggested that employees with high levels of social comparison orientation will show a stronger feeling of envy when they feel that others have received more help and, in turn, reduce their ICB to maintain a balance. 

### 5.1. Theoretical and Practical Contributions

Our findings make several contributions to helping the literature and social comparison theory. First, previous studies about the positive effects of employees receiving help are typically explained using social exchange theory [4], and individuals receiving more help tend to engage in more citizenship behaviors [6]. This stream of research based on social exchange theory to explain employees receiving help ignores the specific context in the organization and only focuses on the absolute value of receiving help [3,31]. This study adopts the new perspective of social comparison [8] to explore the impact of upward social comparison of received help on employees’ psychology and behavior in the team. It is a further expansion of, and supplement to, the previous research on the effects of employees’ absolute value of received help based on social exchange theory [4], which further enriches the research on employees receiving help in the workplace.

Second, we extend the knowledge of the negative consequences of receiving help in the specific context of the team. By exploring envy as an underlying psychological mechanism that helps explain the influence of upward social comparison of received help on employees’ workplace outcomes, we challenge the assumption that all employees will repay received preferential treatment and that receiving help always produces positive effects [16]. These results indicate that the effect of receiving help is influenced by the specific work situation [3]. In the team, those employees who get more help are more likely to gain more resources and achieve good performance. Therefore, this upward social comparison of received help may evoke envy because they perceive the resources as being accumulated by these coworkers, thus reducing their citizenship behaviors to preserve their advantages and resources. This exploration adds to the emerging literature that contends that emotions are crucial to understanding the receiving help phenomena [31]. Moreover, our research enriches scholarly understanding of the antecedents of envy by pointing out that comparing their relative received help in the team is one of the origins of employees’ envy emotion.

Finally, by examining the moderating effects of social comparison orientation on the relationship between the upward social comparison of received help and feelings of envy, we also extend the boundary condition of recipients’ personality characteristics in receiving help and social comparison [17]. Our results suggest that employees high in social comparison orientation might be intensely engaged in social comparisons and be particularly sensitive to learning one’s own standing with others. For employees with higher social comparison orientation, comparing with those who receive more help in the team will stimulate stronger feelings of envy due to the perception of unfair distribution of resources.

The current research also has practical implications. First, our empirical results showed that upward social comparison of received help is positively related to the employee’s feeling of envy and indirectly negatively relates to their interpersonal citizenship behavior, while controlling for overall received help. It indicates that when employees make an upward comparison of received help, they are likely to engender feelings of envy due to the perception of unfair resource allocation, which suggests that leaders in the team should actively guide employees’ attitudes towards receiving help. For example, leaders should emphasize that the work of the team is interdependent, and receiving help is to achieve the common goal of the team better. Second, the team should also create a healthy climate of mutual benefit or establish a balanced reciprocal system [32]. Those who receive more help should try their best to repay the team and help other employees in the team to alleviate others’ feelings of envy.

### 5.2. Limitations and Directions for Future Research

As with any study, our research has several limitations. First, the use of self-reports to assess the constructs in our theoretical model may raise problems of common method variance [29]. However, our research design, meaning that variables were measured at separate points in time (i.e., Time 1, Time 2, and Time 3), should mitigate the concern of common method variance. Future research can also use multi-source measurement methods to verify the model of this study. Second, although we use the three-stage data collection method, it cannot verify the causality of the model. In future research, we can use a scenario experiment or laboratory experiment to improve the internal validity of the model and verify the causality of the model. Third, our findings suggested that individual differences in social comparison orientation may influence the social comparison process in terms of receiving help, such that upward social comparison of received help would elicit higher feelings of envy for those employees with high levels of social comparison orientation. We suggest that future research should focus on other factors that likely impact the effects of receiving help social comparison. For instance, the competitive climate in the team may cause social comparisons to be even more frequent and personally relevant, which further makes organizations a fertile ground for envy [19].

## 6. Conclusions

Based on social comparison theory, the current study aims to extend the research on received help. Results demonstrated that employees are inclined to make social comparisons at the workplace and direct their attitudes and behaviors to their jobs. Specifically, upward social comparison of received help triggered an employee’s feelings of envy, which in turn had a negative relationship with interpersonal citizenship behavior (ICB). Furthermore, we showed that the effects of upward social comparison of received help on envy differed in the employee’s social comparison orientation. We hope our study motivates future research on exploring how social comparison of received help influences essential outcomes in the workplace.

## Figures and Tables

**Table 1:** Descriptive statistics and correlations of variables.

Variables	1	2	3	4	5	6	7	8	9
***Control***									
1. Gender									
2. Age	−0.06								
3. Organization tenure	−0.10 *	0.70 **							
4. Educational background	−0.11 *	0.19 **	0.18 **						
5. Receiving help	0.06	0.13 *	0.15 **	0.08					
***Focal Variables***									
6. Upward social comparison of received help	0.04	0.25 **	0.17 **	0.22 **	0.28 **				
7. Envy	0.07	0.10 *	0.14 **	0.02	0.00	0.28 **			
8. Social comparison orientation	−0.03	0.19 **	0.06	0.03	−0.03	0.03	0.20 **		
9. ICB	−0.00	0.11 *	0.08	−0.04	0.37 **	0.30 **	−0.05	0.05	
Mean	1.52	31.69	5.36	1.72	4.22	4.24	2.77	3.87	4.82
SD	0.50	4.96	2.92	0.69	1.08	1.24	1.12	1.39	1.09

Note: N = 411; * *p* < 0.05; ** *p* < 0.01. The maximum and minimum values for all measurements are 7 and 1, respectively. ICB—interpersonal citizenship behavior.

**Table 2:** Path analysis results.

	Envy		ICB	
Predictor	*γ*	*SE*	*γ*	*SE*
***Control***				
Intercept	2.77 ***	0.05	5.18 ***	0.14
Gender	0.13	0.10	−0.08	0.10
Age	−0.03 *	0.01	0.01	0.02
Organization tenure	0.08 **	0.02	0.00	0.03
Educational background	−0.08	0.07	−0.21 **	0.08
Receiving help	−0.08	0.05	0.31 ***	0.05
***Focal variables***				
Upward social comparison of received help	0.29 ***	0.05	0.24 ***	0.05
Social comparison orientation	0.14 ***	0.04	0.05	0.04
Upward social comparison of received help * Social comparison orientation	0.08 *	0.03		
envy			−0.13 **	0.05

Note: N = 411; * *p* < 0.05; ** *p* < 0.01; *** *p* < 0.001. ICB—interpersonal citizenship behavior; *SE*—standard error.

## Data Availability

Data and analysis codes are openly available at https://osf.io/ksngm/?view_only=ead2c7a35e224be0b2b1a0253d1611fa (accessed on 20 June 2022).
